# Effects of Mixing Ratio and Lactic Acid Bacteria Preparation on the Quality of Whole-Plant Quinoa and Whole-Plant Corn or Stevia Powder Mixed Silage

**DOI:** 10.3390/microorganisms13010078

**Published:** 2025-01-03

**Authors:** Chao He, Qian Li, Huaidong Xiao, Xuchun Sun, Zepeng Gao, Yuan Cai, Shengguo Zhao

**Affiliations:** 1 College of Animal Science and Technology, Gansu Agricultural University, Lanzhou 730070, China; h_5157_2024@163.com (C.H.); li10260101@163.com (Q.L.); 2 Provincial R&D Institute of Ruminants in Gansu, Lanzhou 730070, China; 3 Linxia Hui Autonomous Prefecture Animal Husbandry Technology Promotion Station, Linxia 731800, China; xiaohuaidong_820@163.com (H.X.); sxchun1987@163.com (X.S.); 18809300889@163.com (Z.G.)

**Keywords:** quinoa, corn, stevia, mixed silage, lactic acid bacteria preparation, mycotoxins, bacterial community

## Abstract

Quinoa is the only single plant that can meet all the nutritional needs of human, and its potential for feed utilization has been continuously explored, becoming a prosperous industry for poverty alleviation. In order to further tap the feeding value of whole quinoa, develop quinoa as a feed substitute for conventional crops such as corn, and improve its comprehensive utilization rate, this experiment analyzed the silage quality and mycotoxin content of mixed silage of whole-plant quinoa (WPQ) with whole-plant corn (WPC) or stevia powder(SP) in different proportions, and further improved the silage quality of mixed silage by using two lactic acid bacteria preparations (Sila-Max and Sila-Mix). The quality, microbial population, and mycotoxin levels of quinoa and corn silage, as well as that of the mixed silage of quinoa and stevia, were evaluated using single-factor analysis of variance. The impact of various lactic acid bacteria preparations on the quality of whole-quinoa and whole-corn mixed silage was investigated through two-factor analysis of variance. WPQ and WPC were mixed at the ratio of 5:5 (QB5), 6:4 (QB6), 7:3 (QB7), 8:2 (QB8), 9:1 (QB9) and 10:0 (QB10). SP was mixed with WPQ at the supplemental levels of 0.2% (QB10S2), 0.4% (QB10S4), 0.6% (QB10S6), 0.8% (QB10S8) and 1.0% (QB10S10). After 60 days of silage, the silage indexes, the number of harmful microorganisms, and the mycotoxin levels were measured, to explore the appropriate ratio of mixed silage. The membership function analysis showed that the quality of mixed silage of WPQ with SP was better, and the optimal addition amount of SP was 0.6%. The results of Max and Mix on the quality improvement test of WPQ with WPC mixed silage showed that the two lactic acid bacteria formulations increased CP and AA content, and reduced NH_3_-N/TN; pH was significantly lower than the control group (*p* < 0.01), and LA was significantly higher than the control group (*p* < 0.01). The microbial count results showed that the addition of lactic acid bacteria preparation significantly reduced the number of molds and aerobic bacteria, and the effect of Mix was better than that of Max. When the mixing ratio was between QB7 and QB10, mold was not detected in the lactic-acid-bacteria preparation groups. Max and Mix significantly reduced the levels of mycotoxins, both of which were far below the range of feed safety testing, and 16S rRNA sequencing revealed that the silage microbiota varied with different mixing ratios and whether lactic acid bacteria preparations were used. Max and Mix increased the relative abundance of Firmicutes, with Mix having a more significant effect, especially in the QB6 (65.05%) and QB7 (63.61%) groups. The relative abundance of *Lactobacillus* was significantly higher than that of the control group (*p* < 0.05). The relative abundance of *Enterobacteriaceae* and *Streptococcus* were negatively and positively correlated with the addition level of quinoa, respectively. Comprehensive analysis showed that adding 0.6% SP to the WPQ and using Mix in mixed silage of WPQ and WPC with the proportion of WPQ no less than 70% had the best silage effect, and was more beneficial to animal health.

## 1. Introduction

With the development of animal husbandry in China, silage has been widely used in ruminants. Silage can maintain the nutritional characteristics of green fodder, which has excellent characteristics such as good palatability, strong digestibility, softness, juiciness, and long-term storage. It is of great significance to develop silage in the north, where crops have a short growth period. Optimization of silage technology can improve forage quality and resource utilization. As a common silage method, mixed silage has the advantages of integrating the advantages of multiple silage materials, making up for the lack of fermentation substrate for some raw materials, improving the quality of single silage, and achieving significant feeding effects [1,2]. Currently, this technology has been widely used.

*Chenopodium quinoa* Willd is an annual dicotyledonous herbaceous plant native to the Andes mountains in South America [3]. It is a resilient crop with high nutritional value, high biological yield, and strong environmental adaptability. In addition, it has antioxidant, anti-inflammatory, antifungal, and other pharmacological activities and can be used as a food and for feeding livestock and poultry [4]. A study reported that the lignin content of quinoa straw is lower than that of corn straw, and the crude protein content is equivalent to that of corn straw [5,6]. The feed conversion efficiency of sweet quinoa is higher than that of corn and barley, and its feed value is higher than that of barley, which is equivalent to corn and oats. A study showed that adding quinoa seeds to Merinos sheep could improve meat quality, in terms of better tenderness [7]. Moreover, adding quinoa to the diet of the Luhua chicken can improve growth performance, slaughter performance, and meat quality, reduce diarrhea rate and mortality, and to a certain extent, can significantly reduce the relative abundance of harmful bacteria [8]. Quinoa straw has an essential application prospect as a high-quality feed for animals.

*Stevia rebaudiana* Bertoni is a perennial herb native to South America, in the composite family. Its roots, stems, and leaves are rich in steviol glycosides, with a sweetness far higher than sucrose. It is rich in multiple trace elements and nutrients. It is a natural sweetener with high sweetness, low heat energy, and with safety and non-toxicity characteristics [9]. Pharmacological studies have shown that stevia glycosides carry out biological activities such as lowering blood sugar and blood pressure, and have anti-inflammatory, anti-tumor, antidiarrheal, antibacterial, and immune regulation functions [10,11,12,13]. The application effect of stevia glycosides in animal feed shows that as a sweetener, stevia glycosides can improve the palatability of feed, increase feed intake and weight gain of weaned piglets, reduce diarrhea in piglets [14], and improve lactation performance and nitrogen utilization for lactating dairy cows [15]. In addition, stevia can improve the hepatic antioxidative status of broiler chickens, through enhancing dietary antioxidant availability [16]. At present, stevia as a natural feed additive has received widespread attention at home and abroad.

The production of high-quality silage mainly depends on the composition of the feed at the time of silage and the application of appropriate silage methods. The principle of silage is to achieve low pH quickly, through lactic acid bacteria fermentation, thus maintaining hypoxia conditions [17]. The quality of silage is contingent upon the microorganisms growing on raw materials and their growth during the fermentation process [18]. The number of active lactic acid bacteria attached to most silage materials is limited [19], and it is difficult for it to become the dominant bacteria during natural fermentation, which often leads to proteolysis and dry-matter loss in the silage. Compared with traditional forage crops, whole-plant quinoa (WPQ) has characteristics such as low carbohydrates and high buffering capacity, and is extremely high in moisture content, which is not conducive to obtaining high-quality silages [20]. Therefore, to improve the quality of silage, the fermentation of dominant bacteria, such as lactic acid bacteria, is often promoted by exogenously adding lactic acid bacteria, rapidly reducing the pH value, inhibiting the growth and multiplication of harmful bacteria such as molds and putrefactive bacteria, and inhibiting the production of mycotoxins [21].

The quality and safety issues of silage are increasingly receiving attention. The feed materials used to prepare silage are susceptible to invasion by various harmful microorganisms such as mold before field harvest, during silage storage, and during feeding, and, under certain conditions, they reproduce in large quantities to produce mycotoxins [22], posing a certain threat to the health of livestock and humans. In most cases, hidden feed mildew is difficult to detect, and long-term ingestion by animals can cause subclinical mycotoxin poisoning [23]. These toxins cannot be degraded in animals, and may also be transferred to animal products [24]. Therefore, mastering the presence and variation of harmful microorganisms and mycotoxins in silage is an essential measure for achieving healthy farming. As a new forage resource, the use of quinoa in silage is rarely studied. In this experiment, two silage combinations were set up, namely, adding different proportions of stevia powder (SP) to the WPQ, and mixing silage with different proportions of WPQ and whole-plant corn (WPC). This study evaluated the quality and safety of the two kinds of mixed silage, and then the quality improvement test was carried out on the mixed combination with poor silage effect, by adding lactic acid bacteria preparation. The suitable mixing combinations and additives were selected in order to provide a reference for the efficient use of quinoa in the feed.

## 2. Materials and Methods

### 2.1. Plant Materials and Silage Additives

Quinoa and corn were planted in Guanghe Experimental Field at Gansu Academy of Agricultural Science (35°25′–35°28′ N, 102°23′–103°51′ E, elevation 1953 m above sea level, annual average temperature 6.6 °C, and annual average precipitation 481.2 mm), China. The two silage materials were simultaneously harvested at the wax-ripening and seed-setting stages, and immediately chopped into 2~3 cm pieces using a crop chopper for preparing silages. The composition of the two silage materials is shown in Table 1.

SP (Season Biotechnology Co., Ltd., Xi’an, China); two mixed preparations of lactic acid bacteria (Sila-Max (lactic acid bacteria ≥ 1 × 10^11^ colony-forming units·g^−1^, dosage: 2.5 mg·kg^−1^, effective lactic acid bacteria 2.5 × 10^5^ cfu·g^−1^ fermentation substrate) and Sila-Mix (total calcium content 25%–29.5%, lactic acid bacteria ≥ 1.8 × 10^6^ colony-forming units·g^−1^, dosage: 1000 mg·kg^−1^, effective lactic acid bacteria 1.8 × 10^3^ cfu·g^−1^ fermentation substrate), (Ralco Animal Nutrition, Marshall, MN, USA)).

### 2.2. Experimental Design and Silage Preparation

Test 1: the quinoa and corn plants were chopped into 2~3 cm pieces for silage preparation. A total of 0.2% (QB10S2), 0.4% (QB10S4), 0.6% (QB10S6), 0.8% (QB10S8) and 1.0% (QB10S10) SP were added to the WPQ, respectively; chopped WPQ and WPC were evenly mixed (fresh weight) at the ratio of 5:5 (QB5), 6:4 (QB6), 7:3 (QB7), 8:2 (QB8), 9:1 (QB9) and 10:0 (QB10). After the silage raw materials were mixed fully and evenly, they were quickly packed into the fermentation barrel transformed from a 20 L round-cap polyethene plastic barrel, compacted and sealed, stored in darkness, and fermented at a constant temperature room for 60 days. There were twelve treatments in total, of which single-quinoa silage was used as the control group, and each treatment was conducted in triplicate. The experimental design is shown in Table 2.

Test 2: in Test 1, the mixed silage of WPQ with WPC or SP showed good silage effect, especially the mixed silage of WPQ and SP (data support validation in this paper). The second experiment was conducted to improve the quality of mixed silage, based on the results of the first experiment. The experimental treatments were as follows: Sila-Max(Max) and Sila-Mix(Mix) were added to six mixed-silage groups of WPQ and WPC, respectively, named the Laa group and the Lai group. No-additive-treatment was used as the control group (Lac). According to the instructions for using the additives, Max powder was dissolved in ultrapure water and evenly sprayed on the raw materials, using a micro spray. Mix particles were evenly sprinkled onto the raw materials of silage, and the same amount of distilled water was used in the control group. Each treatment was conducted in triplicate. The experimental design is shown in Table 3.

### 2.3. Silage Quality Analysis

The sensory evaluation of silage was performed according to the German DLG Silage Sensory Scoring Standard. Considering odor, texture, and color as three evaluation indexes, the scores were divided into four grades: excellent (16–20 points), fair (10–15 points), medium (5–9 points), and corrupt (0–4 points).

A fermentation sample was thoroughly mixed with sterile deionized water at the ratio of 1:9, and extracted in a refrigerator at 4 °C for 24 h. Then, the silage extract was prepared by filtration with four layers of gauze and qualitative filter paper, which was used to determine the fermentation indexes [25]. The pH of the extraction solution was determined with a pH meter. The concentrations of organic acid were measured using high-performance liquid chromatography, as described by Tian et al. [26]. The content of ammonia nitrogen (NH_3_-N) was determined using the phenol–sodium hypochlorite colorimetric method [27]. Crude protein (CP) content was determined using the Kjeldahl method [28].

### 2.4. Determination of Microbial Quantity and Mycotoxin Content

Under sterile conditions, 25 g fresh silage samples was weighed, mixed with 180 mL sterile saline water, and oscillated. After an appropriate dilution gradient was obtained through multiple dilutions, the plate dilution method was used to measure the number of colonies of harmful microorganisms in the mixed silage [29]. The CFU of aerobic bacteria and mold were counted on plates with nutrient agar and potato dextrose agar.

The concentrations of aflatoxin B1 (AFB1), zearalenone (ZEN), deoxynivalenol (DON), ochratoxin A (OTA), and T-2 toxins in each mixed-silage sample were determined using enzyme-linked immunosorbent assay (ELISA), as per the manufacturer’s instructions (Beijing Solarbio Technology Co., Ltd., Beijing, China). The results were compared with the allowable limits of mycotoxins mentioned in Chinese Feed Hygiene Standards, to evaluate the contamination of the feed. Among them, aflatoxin B1 ≤ 30 μg·kg^−1^, zearalenone ≤ 1000 μg·kg^−1^, emesis toxin ≤ 5000 μg·kg^−1^, ochratoxin A ≤ 100 μg·kg^−1^, and T-2 toxin ≤ 500 μg·kg^−1^.

### 2.5. Bacterial Community Analysis

According to the manufacturer’s instructions, a Bacterial DNA Kit DP812 (Tiangen Biotech Co., Ltd., Beijing, China) was used to extract DNA from the sample of the fresh whole quinoa and whole-corn mixed silage. The concentrations of extracted DNA samples were detected using a 1900403S Microplate Reader (Thermo Fisher Scientific Inc., Carlsbad, CA, USA). The V3-V4 region of the 16S rDNA gene was amplified by PCR using the universal primers 338F (5′-ACTCCTACGGAGGGGCAGCA-3′) and 806R (5′-GACTACHVGGGTWTCTAAT-3′) to characterize the mixed-silage microbiota of the whole quinoa and whole corn. The PCR products were purified by the magnetic beads (VAHTSTM DNA Clean Beads) method. The 1.8% agarose gels were used to verify the amplified PCR products. The built library was inspected by the Qsep-400 method, and then sequenced on the Illumina novaseq6000 platform (Illumina, San Diego, CA, USA).

The Trimmomatic (v0.33) software was used to filter the raw reads obtained by sequencing, and then cutadapt (1.9.1) software was used to identify and remove the primer sequence to obtain clean reads. The Usearch (v10) software was used to splice the clean reads of each sample through overlay, and then to filter the length of the spliced data according to the length range of different areas. The UCHIME (v4.2) software was used to identify and remove the chimeric sequence to obtain the final effective data. The QIME2 (v2020.6) software was used to evaluate the alpha diversity indexes (Chao1, Ace, Shannon, Simpson) to analyze the species richness and diversity within individual samples, and sample Rarefaction curves and Shannon curves were plotted. Beta diversity analysis compared the similarity of species diversity of different samples. Principal coordinates analysis (PCoA), unweighted pair group method with arithmetic mean (UPGMA) and analysis of similarities were performed at the corresponding distances, according to the distance matrix.

### 2.6. Calculations

The membership-function value method in Fuzzy mathematics was used to comprehensively evaluate the selected indicators of each treatment (silage quality indicators, microbial quantity and mycotoxin content of silage in Test 1), calculate the membership function value of each indicator, and sum and take the average value. Finally, results were ranked according to the average value. The higher the average value, the higher the ranking, and the higher the comprehensive score. The formula for calculating the membership function is as follows:

(1) $$X_{(µ)\mathrm{positive}}=\frac{X-Xmin}{\;Xmax-Xmin}$$

(2) $$X_{(µ)\mathrm{negative}}=1-\frac{X-Xmin}{Xmax-Xmin}$$

In the formula, X(µ) is the specific membership value of a certain indicator, *X* is the measured value of a certain indicator, and *Xmax* and *Xmin* are the maximum and minimum values of the indicator in all treatments, respectively. If the selected indexes were positively correlated with the fermentation quality of silage, they were calculated by Formula (1); if there were a negative correlation, they were calculated using Formula (2).

### 2.7. Statistical Analysis

All statistical data were statistically processed using Excel software (Microsoft Corporation, Redmond, WA, USA) and SPSS (SPSS v 26.0, SPSS, Inc., Chicago, IL, USA) software. The data on silage quality, microbial quantity and mycotoxin content of WPQ and WPC, SP were analyzed by One-way ANOVA. Two-way ANOVA was used to evaluated the effect of the different lactic acid bacteria preparations on the quality of mixed silage of WPQ and WPC. Tukey’s multiple comparison method was used to determine the statistical difference between the mean values. Significance was defined at *p* < 0.05, and high significance was defined at *p* < 0.01.

## 3. Results

### 3.1. Sensory Evaluation of Mixed Silage

After 60 days of silage treatment, the barrels were opened. According to the German Agricultural Society (DLG) scoring standard, the sensory scores of the mixed silage were obtained in terms of smell, color, and texture. The sensory score and rating results are shown in Table 4. It can be seen from Table 4 that QB7, QB8 and QB10S4 have an obvious aroma, accompanied by a bread aroma; in terms of color, QB9 has the most obvious advantage, followed by QB10, QB10S10 and QB10S8. However, QB5, QB6, QB7 and QB10S10 have good texture and a slightly damaged stem and leaf structure. Overall, QB7, QB8, QB5, QB10S10 and B10 have higher evaluation than other groups.

### 3.2. Analysis of Silage Quality and Microbial Quantity of Mixed Silage

The quality characteristics of quinoa silage under different mixed treatments are shown in Table 5. In the mixed silage of WPQ and WPC, the pH of mixed silage in QB5~QB8 showed a significant increasing trend (*p* < 0.05), and the lactic acid content in QB6~QB10 groups was significantly higher than that in the QB5 group (*p* < 0.01); AA content showed a downward trend, and the content of CP increased significantly (*p* < 0.01) with the increasing proportion of quinoa. The ratio of ammonia nitrogen to total nitrogen is an important index to measure dietary protein preservation, and in this mixed-treatment group, there was no significant decrease with the increase of quinoa proportion (*p* > 0.05).

When different proportions of SP were added to WPQ, the CP content was significantly higher than that of the mixed-silage group of WPQ and WPC (*p* < 0.01). The ratio of ammonia nitrogen to total nitrogen reached the lowest in the QB10S8 group, and in QB10S2, QB10S4, QB10S6, and QB10S10 it was higher than that in the mixed-silage groups of WPQ and WPC. The content of LA was significantly higher than that of the mixed-silage groups of WPQ and WPC (*p* < 0.01). But the content of AA of QB10S2~QB10S8 was significantly lower than that of QB5~QB7 (*p* < 0.01).

**Figure 1:**
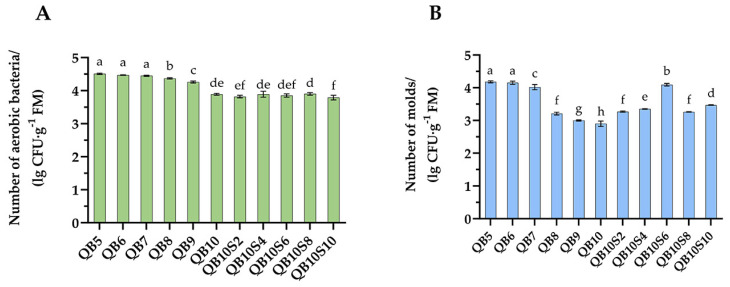
The number of aerobic bacteria (**A**) and molds (**B**) in mixed silage (Test 1). The different lowercase letters (a–h) above the column indicate significant differences among treatments (*p* < 0.05).

As shown in Figure 1A, the number of aerobic bacteria in groups QB7~QB10 was significantly decreased (*p* < 0.05), and the number of bacterial colonies ranged from 3.89 to 4.45 lg cfu·g^−1^. There was no significant difference in the number of aerobic bacteria between the mixed-silage groups of WPQ and SP (*p* > 0.05), but it was significantly lower than that of WPQ and WPC mixed silage (*p* < 0.05), and the number of bacterial colonies ranged from 3.82 to 3.90 lg cfu·g^−1^. The colony count of QB10S10 group was the lowest, at only 3.79 lg cfu·g^−1^.

As shown in Figure 1B, the number of molds in groups QB6~QB10 was significantly decreased (*p* < 0.05), and the number of colonies ranged from 2.90 to 4.15 lg cfu·g^−1^. The number of molds in mixed silage of WPQ and SP was significantly higher than that in mixed silage of the QB9 and QB10 groups (*p* < 0.05), and the number of colonies ranged from 3.26 to 4.09 lg cfu·g^−1^, while the number of colonies in the QB10 group was the lowest, at only 2.90 lg cfu·g^−1^.

### 3.3. Content of Five Mycotoxins in Different Mixed-Silage Treatments

The result of the mycotoxin test for different mixed-silage treatments are shown in Table 6 and Table 7. In two different mixed-silage treatments of quinoa, the detection rates of AFB1, ZEN, DON, OTA, and T-2 toxin in each group were 100%. The contents of ZEN, DON, OTA and T-2 toxins were all within the limit range stipulated by the national feed hygiene standard, while AFB1 exceeded the limit only in the mixed silage of WPQ and WPC, occurring in the two mixing ratios of 50% and 60% quinoa.

**Figure 2:**
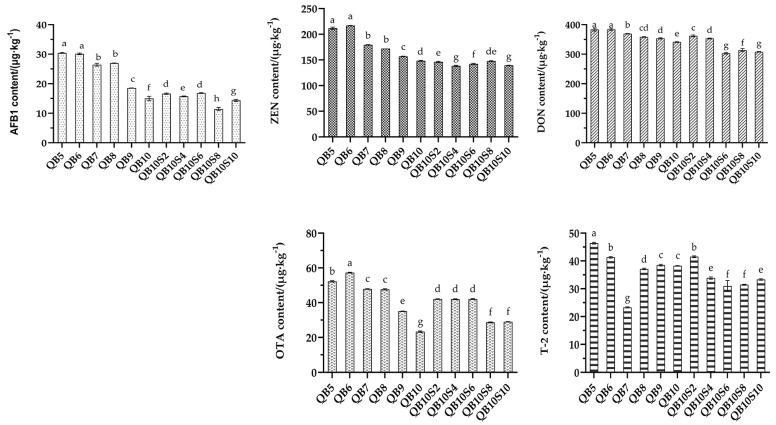
Changes in five mycotoxins’ content in two types of quinoa mixed silage (Test 1). The different lowercase letters (a–h) above the column indicate significant differences among treatments (*p* < 0.05).

Changes in five mycotoxins’ content in two types of quinoa mixed silage are shown in Figure 2. The AFB1 content in the mixed silage of WPQ and SP was significantly lower than that in the mixed silage of WPQ and WPC (*p* < 0.05). Among them, the AFB1 content decreased with the increase in quinoa-addition ratio, and reached the lowest level in the single-quinoa silage (QB10), while reaching the lowest level with the addition of 0.8% SP (QB10S8). Except for T-2 toxin, which showed the lowest content in the QB7 group, the other three toxins, ZEN, DON, and OTA, showed similar situations to AFB1; that is, the toxin content decreased with the increase in quinoa-addition ratio. The toxin content in mixed silage of WPQ and SP groups showed different changes. The trend in ZEN changes among all groups was not significant, but it was significantly lower than that of single-quinoa silage (QB10) (*p* < 0.05). DON was lowest in the QB10S6 group. The content of OTA in each group was significantly higher than that of single-quinoa silage (QB10), and the lowest content was realized in QB10S8 and QB10S10 groups like T-2 toxin.

### 3.4. Comprehensive Evaluation of Membership Functions of Different Types of Quinoa Mixed Silage

The fuzzy membership-function value method was used to comprehensively evaluate the silage quality index, microbial quantity and mycotoxin content of the WPQ and WPC, and the WPQ and SP silage. The larger the calculated function value is, the higher the comprehensive score of the mixed silage will be, which means that the silage quality of the mixed treatment is better, and vice versa. From Table 8, it can be seen that in the mixed silage of WPQ and WPC, when the addition amount of WPQ reaches 90% or more, the membership-function value reaches the maximum (0.5496, 0.6195), which is 41.18% and 47.81% higher than QB5, respectively. In the mixed silage of WPQ and SP, when the addition amounts of SP were 0.6% and 0.8%, the membership-function values were the highest (0.6210, 0.6207), and increased by 20.40% and 20.36%, compared to QB10S2, respectively. Comprehensive analysis shows that among all mixed treatments, QB10S6 has the highest membership-function value (0.6210) and QB5 has the lowest membership-function value (0.3233). Moreover, after adding SP to the WPQ, the overall ranking of comprehensive scores is higher than that of the mixed silage after adding WPC. The ranking of membership-function values of each group is as follows: QB10S6 > QB10S8 > QB10 > QB9 > QB10S4 > QB10S10 > QB7 > QB10S2 > QB8 > QB6 > QB5.

### 3.5. Effect of Additives on Quality of Mixed Silage

The above membership-function analysis results show that the silage quality of the WPQ and WPC mixed silage is slightly lower than that of the WPQ and SP mixed silage. Therefore, a two-factor (additive × mixing ratio) method was adopted to improve the silage quality by adding two lactic acid bacteria preparations, Sila-Max and Sila-Mix. The analysis of mixed-silage quality is shown in Table 9. The primary-effect analysis of different factors revealed that the different mixing ratios had a very significant effect on the pH value and the CP, LA, and AA contents of silage (*p* < 0.01), but not on NH_3_-N/TN (*p* > 0.05). The effect of additives on the pH value and NH_3_-N/TN and LA contents of silage was very significant (*p* < 0.01), and that on the CP and LA contents was significant (*p* < 0.05). The interaction between the two factors only had a very significant effect on the pH value of various treatments (*p* < 0.01), but not on the contents of CP, NH_3_-N/TN, LA and AA (*p* > 0.05).

Under the same mixing ratio, the pH value of the Sila-Max and Sila-Mix groups were significantly lower than those of the control groups (*p* < 0.01). The content of LA was significantly higher than those of the control groups (*p* < 0.01). The content of CP and AA was higher than that of the control groups, while NH_3_-N/TN was lower than that of the control groups, and there was no significant difference among most treatments (*p* > 0.05). No significant difference was observed between the Sila Max and Sila Mix groups (*p* > 0.05). At the same additive level, with the increase in the proportion of quinoa, the pH value and NH_3_-N/TN decreased, while the contents of CP, LA and AA increased, and only a few treatments exhibited significant results.

The microbial quantity analysis revealed that the number of colonies of molds and aerobic bacteria in the control group was the highest under the same mixing ratio and significantly higher than that in additive groups (*p* < 0.01). Among them, the number of colonies of aerobic bacteria in the QB6 and QB8 groups exhibited nonsignificant difference between the Sila-Max and Sila-Max groups (*p* > 0.05), whereas in other mixed groups, the number of colonies of aerobic bacteria in the Sila-Max group was significantly lower than that in the Sila-Max group (*p* < 0.01). In the QB7~QB10 groups, mold colonies were not detected in the Sila-Max and Sila-Mix groups. At the same additive level, the number of colonies of molds and aerobic bacteria significantly decreased with the increase in the proportion of quinoa (*p* < 0.01). According to the comprehensive analysis, the different mixing rations and additives exhibited a very significant impact on the number of colonies of molds and aerobic bacteria (*p* < 0.01), and for different mixing rations and additives a very significant interaction was observed between the two factors (*p* < 0.01).

### 3.6. Effect of Additives on Mycotoxins of Mixed Silage

**Figure 3:**
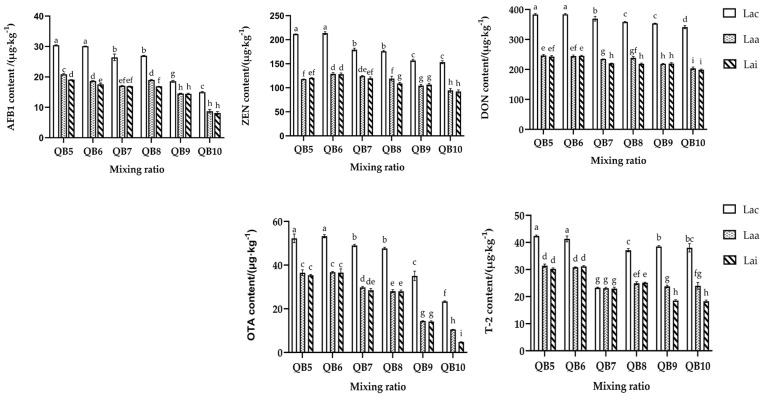
Effect of additives on mycotoxins in mixed silage of WPQ and WPC (Test 2). The different lowercase letters (a–i) above the column indicate significant differences among treatments (*p* < 0.05).

As shown in Figure 3, under the same mixing ratio, the mycotoxin content in the Sila-Max and Sila-Mix groups was lower than that in the control groups, and the difference among most treatment groups was significant (*p* < 0.05). In addition, the difference among various levels of additives was slight, and most of the indicators did not reach a significant level (*p* > 0.05). At the same additive level, the mycotoxin content decreased with the increase in the proportion of quinoa in the mixed silage, and only a few treatment groups did not reach a significant level (*p* > 0.05).

The AFB1 content in the single-quinoa silage group (QB10) was the lowest. Among the blank-treatment groups, the AFB1 content in the LacQB10 group was significantly lower than that in other mixed groups (*p* < 0.05), and the AFB1 content in the LaaQB10 and LaiQB10 groups was lower, by 41.83% and 45.76%, respectively, than that in the LacQB10 group (*p* < 0.05). DON and OTA contents exhibited similar results. After Sila-Max and Sila-Mix were added, the mycotoxin content in the control group decreased by 40.31%, 41.72% and 54.93%, 79.50%, respectively. ZEN exhibited no significant downward trend among the groups (*p* > 0.05). Among the blank-treatment groups, the LacQB9 and LacQB10 groups exhibited the lowest content. The LaaQB10 and LaiQB10 groups were the lowest among the additive-treatment groups. T-2 toxin content was the lowest in the QB7 group.

### 3.7. Changes in Bacterial Community Composition of Mixed Silage of WPQ and WPC

A total of 3,887,362 pairs of reads were obtained by high-throughput sequencing of the control groups and the lactic-acid-bacteria preparation groups of the WPQ and WPC mixed silage. A total of 3,880,219 pieces of clean reads were generated after the quality control and splicing of double-ended reads, and at least 44,495 pieces of clean reads were generated for each sample, with an average of 71,856 pieces of clean reads.

The number of OTUs for each sample was obtained by clustering high-quality sequences at the 97% similarity level, using Usearch (v11) software. The OTU clustering results for each treatment are shown in Table 10. In mixed silage, the OTU of Lac groups and Lai groups did not change significantly with the increase in the proportion of WPQ, but the Laa groups showed an increasing trend. In the same mixing ratio, except for QB8, the number of OTUs in the Laa groups were higher than that in the Lac groups. The number of OTUs in the Lai groups were higher than that in the Lac groups only in QB5, QB9 and QB10. The number of OTUs in the Laa groups were higher than that in the Lai groups.

**Figure 4:**
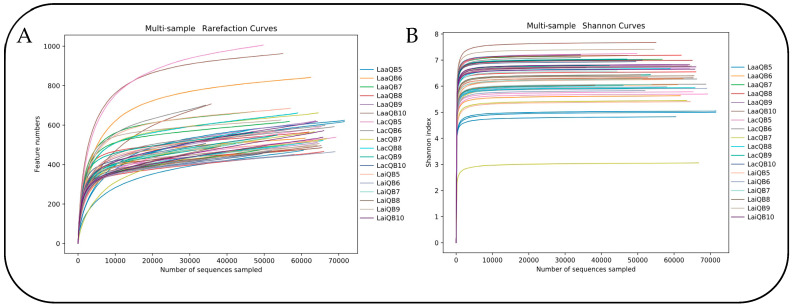
(**A**) Multi-sample rarefaction curves. (**B**) Multi-sample shannon curves (Test 2).

The dilution curve can reflect the species diversity of the sample and indirectly reflect the species richness of the sample. The Shannon curve was also used to reflect the microbial diversity of each sample at different sequencing quantities. As the number of sequences increased, the curve tended to reach a plateau, indicating that the emergence rate of new species gradually decreased and the sequencing coverage was saturated (Figure 4).

The statistical analysis results of the alpha diversity index are shown in Table 11. The Shannon index showed that the bacterial community diversity in the Lac, Laa and Lai groups was not significantly increased with the increasing proportion of WPQ in mixed silage (*p* > 0.05). Under the same mixing ratio, the bacterial diversity of the Laa group and the Lai group with the mixing ratio of QB7, QB8 and QB9 was higher than that of the Lac group, but the difference between the Laa group and the Lai group was not significant. The regularity of the Simpson index variation is not obvious, and the bacterial diversity of samples cannot be accurately analyzed. As can be seen from the Chao1 index and the ACE index, with the increase in quinoa-addition ratio, the species richness of the Lac group is generally declining, while that of the Laa and Lai groups is generally increasing. When the mixing ratio is QB9 and QB10, the addition of Laa and Lai can increase the species richness of the Lac group.

**Figure 5:**
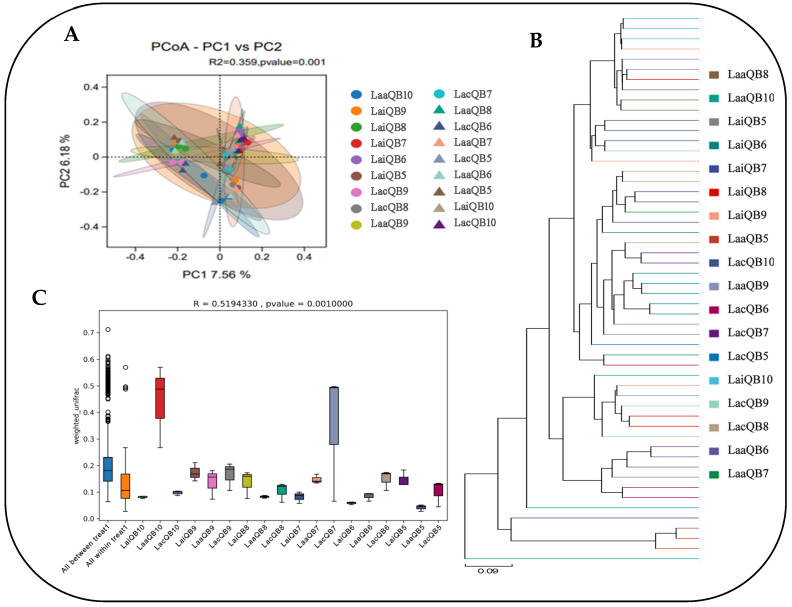
Beta diversity analysis. (**A**) Principal coordinates analysis (PCoA). (**B**) Unweighted Pair group Method with Arithmetic (UPGMA) Mean. (**C**) ANOSIM analysis box plot (Test 2).

The Beta diversity index analysis is shown in Figure 5. The PCoA analysis results, based on the OTU level, showed that the contribution values of PC1 and PC2 to the differences between samples are 7.56% and 6.18%, respectively (Figure 5A). According to UPGMA cluster analysis, samples of each group showed basically independent distribution, indicating that there were differences in bacterial structure and diversity in WPQ and WPC mixed silage (Figure 5B). ANOSIM similarity analysis results, R = 0.519 > 0, further indicated that the difference between groups was greater than the difference within groups, and *p* value = 0.001 < 0.05; the result was significant, indicating high reliability of the test (Figure 5C).

### 3.8. The Effect of Lactic Acid Bacteria Preparation on Microbial Composition of Mixed Silage

**Figure 6:**
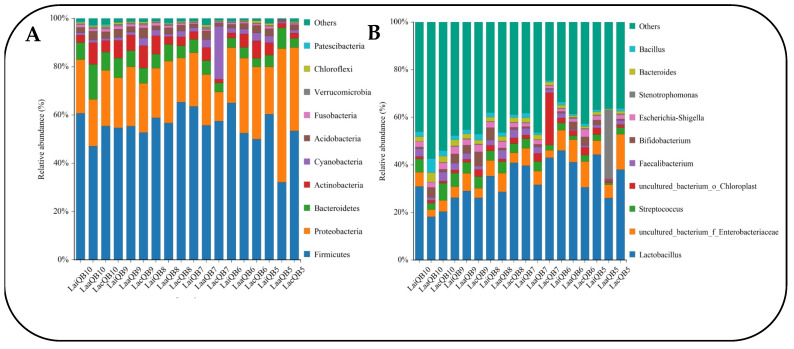
Column diagram of horizontal distribution between groups. (**A**) Composition of bacteria community at phylum level. (**B**) Composition of bacteria community at genus level (Test 2).

At the taxonomy level, a total of 28 phyla, 63 classes, 152 orders, 262 families, 530 genera and 600 species were identified. The top 10 species in terms of relative abundance of mixed silage microorganisms at the phylum and genus levels are shown in Figure 6.

The phylum level mainly includes Firmicutes, Proteobacteria, Bacteroidetes, Actinobacteria, and Cyanobacteria. Among them, the relative abundance of Firmicutes was the highest among the treatment groups (32.20%~65.30%), followed by Proteobacteria (12.08~55.37%) (Figure 6A). At the same time, it can be seen from the figure that after adding Mix (Lai group) to silage with different mixing ratios, the relative abundance of Firmicutes increased, compared with that of Lac groups, while the effect of adding Max on the relative abundance of Firmicutes was not significant. In the non-additive treatment groups (Lac), increasing the proportion of quinoa resulted in an overall upward trend in the relative abundance of Firmicutes, reaching the highest (65.30%) at LacQB8. On the contrary, the relative abundance of Proteobacteria decreases with the increase in quinoa ratio. In addition, the relative abundance of Bacteroidetes and Actinobacteria in each treatment group was 3.56–14.49% and 1.51–9.22%, respectively, and the addition of Max and Mix had no significant effect on the relative abundance of the two.

At the genus level, the composition structure of bacterial genera in different mixed groups is basically the same, but there are certain differences in species richness. Among them, *Lactobacillus* is the dominant bacteria in all mixed groups (relative abundance 18.20~46.04%), followed by *Enterobacteriaceae* (relative abundance 3.00~14.88%) and *Streptococcus* (relative abundance 0.79~7.23%). In addition, it also includes *uncultured_bacterium_o_Chloroplast*, *Faecalibacterium*, *Bifidobacterium*, *Escherichia-Shigella*, *Stenotrophomonas*, *Bacteroides*, *Bacillus*, and some unclassified bacterial species (Figure 6B). Except for the QB7 and QB8 groups, adding Max and Mix can increase the relative abundance of *Lactobacillus* in the Lac group, and the effect of Mix is more significant. In contrast, the effect of lactic acid bacteria preparation on *Enterobacteriaceae* was not significant, but its relative abundance decreased as the proportion of quinoa in mixed silage increased. The relative abundance of *Streptococcus* shows the opposite-change rule, that is, it increases with the increase in quinoa proportion. When the mixed proportion is B10, it reaches the highest level (7.23%). Lactobacillus preparation can also improve its relative abundance.

## 4. Discussion

### 4.1. Effect of Mixed Silage of WPQ and WPC or SP on Silage Quality

The odor, texture, and color of silage are affected by not only the metabolic end-products of fermentation, but also the raw materials. In production, silage quality is generally quickly and directly assessed through sensory evaluation. In this study, when different proportions of WPC or SP were added to WPQ for 60 days of silage fermentation, no corruption was found, and the sensory evaluation score was good or between good and excellent. This indicated that adding WPC or SP to WPQ for mixed silage could obtain silage with good sensory properties.

The two types of mixed silage in this experiment have good quality. CP is an important index for evaluating the nutritional quality of silage. The higher the content, the higher the nutritional quality, and vice versa. As a high-protein feed, the CP content of quinoa increased when it was mixed with stevia silage. In addition, stevia is a non-toxic sugar cash crop with high sweetness and low calories [30], and the sugar content of silage is also an important factor affecting the success of silage [31]. The high sugar content in mixed silage can provide sufficient substrate for lactic acid bacteria fermentation, thus promoting the decrease in pH value, inhibiting the propagation of spoilage bacteria, and reducing the loss of CP and other nutrients [32]. Runbo Luo et al. [33] showed that with the increase in sweetness, most nutrients (dry matter and crude protein) are preserved, and water-soluble carbohydrates are fully utilized to promote fermentation, resulting in a decrease in pH and a significant increase in lactic acid content and ratio of lactic acid to acetic acid, indicating a shift to homofermentation. In addition, adding an appropriate amount of stevia can improve the sweetness of feed, thus improving the palatability of feed and increasing the feed intake of livestock. Kung et al. believed that the formation of ammonia nitrogen was related to the degradation of CP, while NH_3_-N/TN reflected the degradation degree of CP [34]. The higher the percentage of NH_3_-N/TN in silage, the more protein decomposition of the feed, and when the ratio is lower than 10%, it can be identified as high-quality silage [35]. In this study, the NH_3_-N/TN content of each treatment was lower than 10%, indicating that the two mixed silages reached the standard of high-quality silage, but, at the same time, there were different degrees of CP hydrolysis.

The accumulation of lactic acid in the silage process promotes the decline in pH value, which is the premise to ensure the quality of silage. It is generally believed that the pH value of high-quality silage is between 3.8 and 4.2 [36]. In the mixed silage of WPQ and WPC, pH showed an increasing trend with the increase of quinoa, which may be due to the fact that quinoa has a lower carbohydrate content compared with traditional crops, such as wheat and corn [37], which cannot provide sufficient fermentation substrate for the growth of lactic acid bacteria, resulting in the reduction of lactic acid production and the eventual increase in the pH. When stevia was added to quinoa, the overall pH of the mixed silage was slightly higher than that of WPQ and WPC mixed silage, and the lactic acid content confirmed this result. However, in this study, the pH of the two kinds of quinoa mixed silage was within the pH range of high-quality silage, indicating that adding WPC or SP to quinoa could improve the fermentation quality of quinoa silage.

Silage fermentation is a dynamic process mediated by microorganisms competing with each other. Good microbial community composition is the key to producing high-quality silage. As a critical microorganism in the silage production process, lactic acid bacteria are the drivers of lactic acid fermentation [20], whereas molds are the main harmful microorganisms that spoil the silage, produce mycotoxins, and deteriorate the feed quality [38]. In this study, the order of magnitude of mold and aerobic bacteria in each mixed-treatment group were all within the prescribed range of China’s feed hygiene standards, because the low pH value of mixed silage inhibited the propagation of harmful microorganisms such as mold and aerobic bacteria. In addition, the number of mold and aerobic bacteria decreased with the increase in the proportion of quinoa, which may be related to the fact that quinoa plants have saponins with antioxidant, antibacterial, and other pharmacological activities [39]. Antibacterial studies on saponins have shown that their antibacterial effect is concentration-dependent and positively correlated [40]. It has been proved that quinoa saponins have the same antibacterial activity as the Chinese medicinal materials, saponins.

### 4.2. Effect of Mixed Silage of WPQ and WPC or SP on Mycotoxin Content

Mycotoxin is a secondary metabolite produced by molds under appropriate conditions [41]. Due to the widespread existence of toxin-producing fungi, mycotoxins are present in several livestock and poultry feeds [42]. The contamination of silage with molds and mycotoxins and related safety concerns of animal feeding have attracted attention. In most cases, invisible feed mildew is difficult to find, and long-term ingestion of animals will cause subclinical mycotoxin poisoning, such as reduced production performance, immune suppression, reproductive dysfunction, liver and kidney organ damage, and even the death of animals [43]. Moreover, these toxins do not degrade in animals, and may be transferred to animal products [24]. Sensory evaluation in this study demonstrated that each mixed silage had no mildew, while the results of five toxins were all positive. Except for AFB1, which was slightly exceeding the standard in individual groups, the other toxins were far lower than the national feed hygiene standard of China. This proved that the silage without obvious mold contamination could also have the some levels of mycotoxins [44]. At the same time, it also shows the importance of silage safety detection for animal health.

This study demonstrated that the level of five mycotoxins decreased by various degrees with the increase in the proportion of quinoa in the mixed silage. This phenomenon may be related to the number of colonies of toxin-producing molds. The low pH value of mixed silage inhibited the mildew activity, and thus the mycotoxin production decreased accordingly. This study found that the overall mycotoxin content of WPQ and SP mixed silage was lower than that of WPQ and WPC mixed silage, which may be caused by different types of silage materials [45].,AFB1 and OTA are the metabolites produced by Aspergillus [46]. Their content decreased with the increase in the proportion of quinoa; however, the result was the opposite in the case of corn. This is because most fungi, mainly Aspergillus and Fusarium, easily infect and colonize corn crops [47], and Aspergillus is more suitable to surviving under low water activity and high temperature than Fusarium [48]. Fusarium may produce the other three mycotoxins, and temperature and water activity will affect its survival [49]. Thus, feeding quinoa-added silage can improve the health of animals. In addition, the content of mycotoxins is also affected by the environmental conditions during the harvesting of silage materials [45]. The content of DON is positively correlated with the average temperature and minimum temperature on the day of harvest of the raw materials of silage [50]. The silage in this study was prepared in the middle of October. The local average temperature was 15 °C, and the lowest temperature was 5 °C. As the temperature was low, it effectively controlled mycotoxin production. Unlike the other four mycotoxins, the average content of DON was the highest, which may be because DON is a trichothecene mycotoxin produced by *Fusarium*, which is not affected by acidic and anaerobic conditions [51].

### 4.3. Effect of Lactic Acid Bacteria Preparation on the Quality of Mixed Silage of WPQ and WPC

The ideal fermentation of silage is dominated by lactic acid bacteria as the dominant microbial community, and a large number of studies have shown that the limited number of lactic acid bacteria attached to the surface of silage raw materials can easily cause harmful bacteria to grow and reproduce, leading to nutrient loss and a decrease in silage quality [52]. Lactic acid bacteria additives are one of the important measures for improving the quality of silage, providing an important guarantee for the preparation of high-quality silage. The study showed that the lactic-acid-bacteria preparation groups effectively improved the fermentation conditions, which was related to the addition of lactic acid bacteria promoting the accumulation of lactic acid during the early stage of silage production and rapidly reducing the pH value of the silage. At the same time, the results showed that the contents of LA and AA in silage with added Max and Mix were significantly higher than those in the control group, while the contents of CP and NH_3_-N/TN increased and decreased, respectively. This is mainly because the lactic acid bacteria contained in Max and Mix additives promoted lactic acid fermentation, significantly increased the lactic acid content, decreased the pH of silage, and effectively inhibited the decomposition of feed protein by harmful bacteria, thereby reducing NH_3_-N/TN content [53]. In addition to the standard components (various types of lactic acid bacteria and bacteria that can produce amylase and cellulase) shared by Mix and Max, 25%–29.5% CaCO_3_ was added to the components. CaCO_3_ can improve the organic acid content and pH value of the silage. The pH of the Sila-Max group was lower than that of the Mix group. On the one hand, Sila-Max exhibited a better effect on the promotion of lactic acid fermentation, but, on the other hand, it may be related to the fact that CaCO_3_ in the Max component neutralizes hydrogen ions in the acidic environment.

In this study, the type of additives, mixing ratio of quinoa and corn straws, and the interaction between the two factors significantly impacted the number of colonies of molds and aerobic bacteria. At the same additive level, the number of colonies of molds and aerobic bacteria exhibited a downward trend with the increase in the proportion of quinoa straw. This was because when quinoa is mixed in corn silage, the lower pH effectively inhibits the propagation of harmful microorganisms in the silage production process. Moreover, after adding Max and Mix to the mixed silage, the number of molds and aerobic bacteria decreased significantly. This is because Max and Mix used in this study belong to composite lactic acid bacteria preparation, which also contains homotypic- and heterotypic-fermentation lactic acid bacteria, which can increase the content of organic acids such as acetic acid while reducing the pH of silage. It also can improve the aerobic stability of silage and inhibit the growth and reproduction of harmful microorganisms such as mold [54]. Moreover, the research results showed that no mold was detected when the quinoa-addition ratio was not less than 70%, so the mixed silage with this ratio and above was safer and more beneficial to the health of livestock. This proved that lactic acid bacteria preparation can significantly improve the fermentation quality of mixed silage [55].

### 4.4. Effect of Lactic Acid Bacteria Preparation on the Content of Mycotoxins in the Mixed Silage of WPQ and WPC

In this study, the content of five mycotoxins in all silage samples did not exceed the limits of China’s feed hygiene standards, and after adding lactic acid bacteria preparation, the mycotoxins were significantly lower than the control group. On the one hand, the use of lactic acid bacteria preparations promotes a decrease in the pH of silage, inhibits mold activity, and reduces the number of toxin-producing molds, resulting in a decrease in fungal toxin production; on the other hand, it may be related to the composition of lactic acid bacteria in the preparation of lactic acid bacteria. At present, a large number of studies have been conducted to delay the proliferation of spoilage bacteria such as yeast and mold in feed by inoculating exogenous lactic acid bacteria, so as to improve the fermentation or aerobic stability of silage [56]. It has been shown that some lactic acid bacteria can degrade or immobilize mycotoxins by binding to the surface of mycotoxins [51,57]. In addition, some lactic acid bacteria can also produce antifungal compounds such as organic acids, carboxylic acids and phenolic compounds, to reduce mycotoxins produced by mold [58]. Some studies have also suggested that some bacteria in silage can also reduce the concentration of fungal toxins, regardless of whether they are inoculated with lactic acid bacteria [59]. The changes in mycotoxin content in this study may also be related to the role played by lactic acid bacteria themselves. But the potential effects and specific mechanisms of the two lactic acid bacteria preparations on mycotoxins still need further exploration. However, it can be proved that adding lactic acid bacteria preparation in the process of silage production can improve the safety of silage, which has a positive role in ensuring the health of ruminants and product safety.

### 4.5. Effects of Lactic Acid Bacteria Preparation on Bacterial Community in Mixed Silage of WPQ and WPC

Bacteria are an important microbial group in the fermentation process of silage, maintaining a large composition of bacterial species. They are mainly divided into two categories: one is beneficial bacteria that dominate silage fermentation, such as *Lactobacillus*, and the other is harmful bacteria that compete with *Lactobacillus* for nutrients and use substrates such as sugar and lactic acid in silage to cause dry-matter loss, protein degradation, and even toxic substances, such as *Fusobacterium*, intestinal bacteria, *Listeria*, *Acetobacter*, etc. [60]. While normal silage fermentation is determined by a few dominant bacteria, a large number of harmful bacteria will cause the silage system disorders. Therefore, it is of great significance to analyze the structural diversity and abundance variation of bacterial flora in the process of silage fermentation for the evaluation of nutrient composition and fermentation quality of silage [61]. Next-generation sequencing has been widely used in the study of silage microorganisms, and can comprehensively and accurately describe microbial community information and accurately measure the composition and relative abundance of various microorganisms in different samples. This study analyzed the bacterial community structure of whole-quinoa and whole-corn mixed silage treated with different lactic acid bacteria preparations, and defined the diversity of bacterial communities in different silage samples from OTU clustering, Alpha/Beta diversity, community composition, etc. The original data were finally obtained through quality-control splicing and the filtering and removal of chimeras. According to the results, the quality sequences of all silage samples were more than 20,000, indicating that the sequencing volume of samples had reached saturation and most of the bacteria were covered, which could adequately reflect the species information of most bacteria in the samples.

Alpha diversity is mainly used to assess the diversity of silage microflora, reflecting species richness, evenness and sequencing depth [62]. The OTU statistical results showed that the number of OTUs in the Laa and Lai groups were higher than that in the Lac group, and the number of OTUs in the Laa group was significantly higher than that in the Lai group among multiple mixing ratios, indicating that both Max and Mix could improve the microbial species of mixed silage, and the species diversity was different with different additive types. According to the Alpha diversity analysis, when the mixing ratio of the Laa and Lai groups were QB7~QB10, the Chao1 index and ACE index were both increased compared with the Lac group, and their species richness was also relatively increased, which was basically consistent with OTU analysis results. The possible reason is that lactic acid bacteria preparation can improve the aerobic stability of silage during the silage process [63].

According to the high-throughput sequencing results, the core flora was analyzed at the phylum level; it was found that all mixed-silage treatment groups were similar in the composition of flora, and the dominant phyla of each group were Firmicutes and Proteobacteria, but the relative abundance of flora in different treatment groups was somewhat different, which was consistent with the results of Wang et al. [64]. Firmicutes belong to Gram-positive bacteria, including most bacteria involved in lactic acid fermentation, which can produce protease, lipase, cellulase and other enzymes to degrade protein, cellulose and other macromolecular substances, providing more substrates for the activities of silage-fermentation microorganisms [65]. Proteobacteria belong to Gram-negative bacteria, including many pathogenic bacteria such as Escherichia coli and salmonella, which compete with lactic acid bacteria for fermentation substrate, resulting in loss of feed protein and increase in ammonia nitrogen content [66]. In this study, the Firmicutes rapidly increased after silage and quickly became the dominant phylum in all mixed-silage groups, and their relative abundance was higher when quinoa was added at a ratio of 70% or more. On the contrary, the relative abundance of Proteobacteria was higher in treatments with a quinoa-addition ratio of 50–60%, compared to other treatments. In addition, the relative abundance of Firmicutes in the additive groups showed an increasing trend compared to the control groups, with the Lai groups having the highest relative abundance. The results showed that the addition of quinoa could improve the fermentation environment of corn silage, and the use of lactic acid bacteria preparation could improve the bacterial community structure, reduce the proportion of Proteobacteria, to a certain extent, and reduce the potential risk of pathogenic bacteria to livestock health in feed. Moreover, when the addition proportion of quinoa in mixed silage was 70% or more, and the lactic acid bacteria preparation was Mix, this was the ideal mixing ratio and additive type.

Further analysis of bacterial community composition at the genus level showed that *Lactobacillus* was the dominant bacteria in all treatments, followed by *Enterobacteriaceae* and *Streptococcus*. *Lactobacillus* is the main beneficial bacteria in silage fermentation, with strong acid resistance [67]. After the two kinds of lactobacillus preparations were treated, the relative abundance of *Lactobacillus* in most treatments was higher than that in the control groups, indicating that the addition of lactobacillus preparations in mixed silage made *Lactobacillus* dominant in the silage system, and it was reported that lactic acid bacteria in silage was mainly *Lactobacillus* [68]. Therefore, a large amount of lactic acid can be produced in the silage process, to reduce pH and inhibit the propagation of harmful microorganisms, which is also the direct cause of the high Firmicutes content in this test. Studies have shown that *Enterobacteriaceae* can use lactic acid produced by silage fermentation to degrade feed protein and produce butyric acid and ammonia nitrogen [66]. In this experiment, the relative abundance of *Enterobacteriaceae* decreased gradually with the increase in the proportion of the whole quinoa, which showed an opposite trend to that of *Streptococcus*. This was because the beneficial lactic acid bacteria such as *Lactobacillus* and *Streptococcus* became the dominant bacteria in the fermentation system, producing a large amount of lactic acid and quickly forming an acidic environment that was sufficient to inhibit the level of *Enterobacteriaceae* and other corrupt bacteria. Thus, it better guarantees the fermentation quality of silage.

## 5. Conclusions

In conclusion, WPQ mixed with WPC or SP could achieve good silage effect; the results of membership-function analysis showed that the quality of WPQ and SP mixed silage was better, and the optimal addition amount of SP was 0.6%. Therefore, the quality improvement test was carried out by adding Max and Mix to the mixed silage of WPQ and WPC. At the same time, various mixing ratios and two additives were analyzed using two-factor primary-effect analyses, which revealed the best mixed-storage mode. Both of the two lactic acid bacteria preparations can improve the quality of mixed silage and reduce the number of microorganisms and mycotoxin content, but, in terms of silage safety, Mix showed better bacteriostatic effect. The mixed silage with the proportion of quinoa as ≥70% and 1000 mg·kg^−1^ Sila-Mix was the best for animal health.

## Figures and Tables

**Table 1:** Chemical composition of silage materials.

Parameter	WPQ	WPC
Dry matter (% FM)	35.50	32.64
Crude protein (% DM)	13.51	7.42
Ether extract (% DM)	7.39	6.09
Crude ash (% DM)	13.20	4.61
Neutral detergent fiber (% DM)	48.37	54.54
Acid detergent fiber (% DM)	31.84	25.01

FM, fresh-matter basis; DM, dry-matter basis; WPQ, whole-plant quinoa; WPC, whole-plant corn.

**Table 2:** Treatments of Test 1.

Material	Treatment										
	QB5	QB6	QB7	QB8	QB9	QB10	QB10S2	QB10S4	QB10S6	QB10S8	QB10S10
WPQ	5	6	7	8	9	10	10	10	10	10	10
WPC	5	4	3	2	1	0	-	-	-	-	-
SP	-	-	-	-	-	-	0.2%	0.4%	0.6%	0.8%	1.0%

WPQ, whole-plant quinoa; WPC, whole-plant corn; SP, stevia powder.

**Table 3:** Treatments of Test 2.

Treatment	Ratio of WPQ and WPC					
	5:5 (QB5)	6:4 (QB6)	7:3 (QB7)	8:2 (QB8)	9:1 (QB9)	10:0 (QB10)
Lac	LacQB5	LacQB6	LacQB7	LacQB8	LacQB9	LacQB10
Laa	LaaQB5	LaaQB6	LaaQB7	LaaQB8	LaaQB9	LaaQB10
Lai	LaiQB5	LaiQB6	LaiQB7	LaiQB8	LaiQB9	LaiQB10

Treatment: Lac, control; Laa, Sila-Max; Lai, Sila-Mix.

**Table 4:** Sensory evaluation of mixed silage (Test 1).

Group	Mildew	Smell		Color		Texture		Composite Scores	
		Description	Average Score	Description	Average Score	Description	Average Score	Total Score	Grade
QB5	No mildew	A faint smell of bread.	11.33	Yellowish-brown.	1.30	The stem and leaf structure are well-maintained, soft and loose.	2.83	15.46	Between good and excellent
QB6	No mildew	A faint smell of bread.	11.33	Medium, yellow, with a hint of green.	1.33	The stem and leaf structure are maintained well, and loose.	2.63	15.29	Between good and excellent
QB7	No mildew	Slightly aromatic.	11.66	Yellowish-brown.	1.30	The stem and leaf structure are maintained well, and loose.	2.63	15.59	Between good and excellent
QB8	No mildew	Slightly aromatic.	11.66	Light yellowish brown, with a hint of green.	1.43	The stems and leaves do not rot, and the texture is soft and loose.	2.50	15.59	Between good and excellent
QB9	No mildew	A faint sour smell.	10.66	Light yellow, light green.	1.56	The stem and leaf structure are well-maintained, and slightly sticky.	2.33	14.55	Good
QB10	No mildew	A faint sour smell.	10.33	Light yellowish brown, with a hint of green.	1.53	The stem and leaf is slightly damaged, and sticky	2.00	15.42	Between good and excellent
QB10S2	No mildew	No butyric acid odor, weak aromatic smell.	10.66	Yellowish-brown.	1.23	The stem and leaf is slightly damaged, and slightly sticky.	2.13	14.02	Good
QB10S4	No mildew	Slightly aromatic.	11.66	Yellowish-brown.	1.20	The stem and leaf structure are maintained well, and loose.	2.43	15.29	Between good and excellent
QB10S6	No mildew	No butyric acid odor, weak aromatic smell.	10.66	Light yellowish brown, with a hint of green.	1.43	The stem and leaf structure are maintained well, and loose.	2.40	14.49	Good
QB10S8	No mildew	No sour odor, weak aromatic smell.	11.00	Yellow with a hint of green,	1.50	The stem and leaf structure are maintained well, and loose.	2.43	14.93	Good
QB10S10	No mildew	A faint smell of bread.	11.33	Yellow-green.	1.53	The stem and leaf structure are well-maintained, soft and loose.	2.60	15.46	Between good and excellent

Please see the composition of groups in Table 2.

**Table 5:** Silage quality of mixed fermentation of WPQ with WPC or SP in different proportions (Test 1).

Groups	Crude Protein (CP)/% DM	pH	NH_3_-N/TN /%	Lactic Acid (LA)/g·kg^−1^ DM	Acetic Acid (AA)/g·kg^−1^ DM
QB5	9.16 E	3.96 D	6.89	20.44 B	2.26 A
QB6	10.10 D	4.02 CD	6.88	21.88 A	2.13 AB
QB7	10.46 CD	4.08 BC	6.21	22.82 A	1.77 BC
QB8	10.65 C	4.10 AB	6.37	22.83 A	1.49 C
QB9	12.90 B	4.13 AB	6.22	22.83 A	1.56 C
QB10	13.39 AB	4.10 AB	6.29	23.01 A	1.84 BC
QB10S2	13.40 AB	4.13 AB	7.66	20.37 B	1.76 BC
QB10S4	13.06 AB	4.15 A	7.00	18.29 C	1.66 C
QB10S6	13.23 AB	4.10 AB	6.87	18.19 C	1.68 C
QB10S8	13.18 AB	4.15 AB	5.59	18.73 C	1.76 BC
QB10S10	13.51 A	4.14 AB	7.10	18.34 C	1.84 BC
SEM	0.28	0.01	0.16	0.36	0.05
*p*-Value	**	**	NS	**	**

The different capital letters in the same column show extremely significant differences at *p* < 0.01. SEM: standard error of mean. ** *p* < 0.01, and NS: no significant differences.

**Table 6:** Result of the mycotoxin test for mixed silage of WPQ and WPC (Test 1).

Item	Quinoa + Corn				
	Aflatoxin B1 (AFB1)	Zearalenone (ZEN)	Deoxynivalenol (DON)	Ochratoxins (OTA)	T-2 Toxin (T-2)
Number of detection groups	18	18	18	18	18
Detection range/(μg·kg^−1^)	14.28~30.60	147.41~217.12	339.34~388.64	22.91~57.47	23.14~46.69
Detection rate/%	100	100	100	100	100
Excessive number	5	0	0	0	0

**Table 7:** Result of the mycotoxin test for mixed silage of WPQ and SP (Test 1).

Item	Quinoa + Stevia				
	Aflatoxin B1 (AFB1)	Zearalenone (ZEN)	Deoxynivalenol (DON)	Ochratoxins (OTA)	T-2 Toxin (T-2)
Number of detection groups	15	15	15	15	15
Detection range/(μg·kg^−1^)	10.89~16.99	137.29~148.51	300.28~365.12	28.37~42.36	29.70~41.81
Detection rate/%	100	100	100	100	100
Excessive number	0	0	0	0	0

**Table 8:** Membership-function value analysis and comprehensive ranking of different type of quinoa mixed silages (Test 1).

Item	Membership-Function Value										
	QB5	QB6	QB7	QB8	QB9	QB10	QB10S2	QB10S4	QB10S6	QB10S8	QB10S10
CP	0.0000	0.2161	0.2989	0.3425	0.8598	0.9724	0.9747	0.8966	0.9356	0.9241	1.0000
Moisture content	1.0000	0.6667	0.5556	0.3333	0.3333	0.1111	0.3333	0.2222	0.1111	0.0000	0.2222
pH	1.0000	0.6842	0.3684	0.2632	0.1053	0.2632	0.1053	0.0000	0.2632	0.0000	0.0526
NH_3_-N/TN	0.3720	0.3768	0.7005	0.6232	0.6957	0.6618	0.0000	0.3188	0.3816	1.0000	0.2705
LA	0.4668	0.7656	0.9606	0.9627	0.9627	1.0000	0.4523	0.0207	0.0000	0.1120	0.0311
AA	0.0000	0.1688	0.6364	1.0000	0.9091	0.5455	0.6494	0.7792	0.7532	0.6494	0.5455
Molds	1.0000	0.9766	0.8750	0.2422	0.0781	0.0000	0.2891	0.3516	0.9297	0.2813	0.4453
Aerobic bacteria	0.0000	0.0556	0.0833	0.1944	0.3472	0.8611	0.9583	0.8611	0.9167	0.8472	1.0000
AFB1	0.0000	0.0168	0.2111	0.1826	0.6258	0.8126	0.7263	0.7753	0.7163	1.0000	0.8463
ZEN	0.0637	0.0000	0.4774	0.5704	0.7636	0.8733	0.9001	1.0000	0.9509	0.8799	0.9884
DON	0.1529	0.1480	0.3006	0.4171	0.4677	0.5984	0.3771	0.4716	1.0000	0.8836	0.0000
OTA	0.1477	0.0000	0.2772	0.2825	0.6544	1.0000	0.4488	0.4497	0.4477	0.8431	0.8340
T-2	0.0000	0.2196	1.0000	0.4010	0.3426	0.3538	0.2113	0.5431	0.6674	0.6479	0.5660
Mean	0.3233	0.3304	0.5188	0.4473	0.5496	0.6195	0.4943	0.5146	0.6210	0.6207	0.5232
Ranking	11	10	7	9	4	3	8	5	1	2	6

**Table 9:** The effect of additives on the quality and microbial quantity of mixed silage (Test 2).

Mixing Ratio	Additive	pH	CP	NH_3_-N/TN (%)	LA (g·kg^−1^ DM)	AA (g·kg^−1^ DM)	Microorganisms (lg CFU·g^−1^ FM)	
							Mold	Aerobic Bacteria
QB5	Lac	3.96 D	9.16 h	6.89 a	20.44 F	2.26 CDE	4.18 A	4.51 A
	Laa	3.84 F	9.57 gh	6.53 ab	27.54 C	2.62 BC	3.21 C	4.35 C
	Lai	3.90 E	9.86 fgh	6.04 ab	27.56 C	2.69 BC	3.13 D	4.24 D
	Mean	3.90 C	9.53 E	6.49 a	25.18 D	2.52 B	3.51 A	4.37 A
QB6	Lac	4.02 C	10.10 fgh	6.88 a	21.88 E	2.13 CDEF	4.15 A	4.47 AB
	Laa	3.90 E	10.47 fg	5.39 b	28.38 ABC	3.67 A	2.81 G	4.25 D
	Lai	3.96 D	10.70 5fg	6.22 ab	27.41 C	3.08 AB	2.73 H	4.20 D
	Mean	3.96 A	10.43 D	6.16 ab	25.89 C	2.96 A	3.23 B	4.31 B
QB7	Lac	4.08 B	10.46 fg	6.21 ab	22.82 DE	1.77 DEF	4.02 B	4.45 B
	Laa	3.80 G	10.93 ef	5.57 ab	28.36 ABC	2.42 BCD	—	4.24 D
	Lai	3.79 G	10.53 fg	6.04 ab	27.63 C	2.22 CDEF	—	3.97 E
	Mean	3.89 C	10.64 D	5.94 ab	26.27 BC	2.14 C	1.34 C	4.22 C
QB8	Lac	4.10 AB	10.65 fg	6.37 ab	22.83 DE	1.49 EF	3.21 C	4.37 C
	Laa	3.79 G	12.08 cd	5.38 b	28.73 AB	1.80 DEF	—	4.06 E
	Lai	3.80 G	11.76 de	5.85 ab	27.87 BC	1.81 DEF	—	3.97 E
	Mean	3.90 C	11.50 C	5.87 ab	26.48 AB	1.70 D	1.07 D	4.13 D
QB9	Lac	4.13 A	12.90 bc	6.22 ab	22.83 DE	1.56 EF	3.00 E	4.26 D
	Laa	3.78 G	13.66 ab	5.44 ab	28.75 AB	1.60 EF	—	3.95 E
	Lai	3.85 F	12.59 bcd	5.39 b	28.03 BC	1.61 EF	—	3.80 G
	Mean	3.92 B	13.05 B	5.68 b	26.53 AB	1.59 D	1.00 E	4.00 E
QB10	Lac	4.10 AB	13.39 ab	6.29 ab	23.01 D	1.84 DEF	2.90 F	3.89 F
	Laa	3.69 H	13.64 ab	5.18 b	29.18 A	1.46 F	—	3.74 H
	Lai	3.77 G	14.14 a	3.85 ab	28.77 AB	1.47 F	—	3.17 I
	Mean	3.85 D	13.72 A	5.77 ab	26.98 A	1.59 D	0.97 E	3.60 F
Main-effect analysis								
*p*	A	**	*	**	**	*	**	**
	B	**	**	NS	**	**	**	**
	A*B	**	NS	NS	NS	NS	**	**

The different small letters in the same column show significant differences at *p* < 0.05; different capital letters in the same column show extremely significant differences at *p* < 0.01. The mean of the B treatments is compared separately. ** means *p* < 0.01, * means *p* < 0.05, NS means no significant differences.

**Table 10:** OTU statistical table (Test 2).

Treatment	Mixing Ratio					
	QB5	QB6	QB7	QB8	QB9	QB10
Lac	566	632	550	551	541	477
Laa	622	688	646	506	575	723
Lai	592	463	526	492	545	578

**Table 11:** The effects of different additive treatments on alpha diversity index (Test 2).

Mixing Ratio	Additive	Index Type			
		Shannon	Simpson	Chao1	ACE
QB5	Lac	6.25	0.95	847.52	914.23
	Laa	4.96	0.89	665.40	647.43
	Lai	6.00	0.91	708.92	684.71
QB6	Lac	6.48	0.96	781.20	770.03
	Laa	5.99	0.93	697.30	701.59
	Lai	5.91	0.93	603.90	708.08
QB7	Lac	4.82	0.81	734.72	776.77
	Laa	6.84	0.97	752.70	865.56
	Lai	6.31	0.94	678.77	810.92
QB8	Lac	6.21	0.94	741.03	874.80
	Laa	6.91	0.98	666.11	910.74
	Lai	6.39	0.96	640.12	772.31
QB9	Lac	6.76	0.97	722.50	886.91
	Laa	6.76	0.97	748.66	975.36
	Lai	6.98	0.98	770.52	980.91
QB10	Lac	7.05	0.98	675.69	909.40
	Laa	6.90	0.98	820.66	876.10
	Lai	6.74	0.98	767.40	974.72
SEM		0.105	0.007	13.67	22.58
*p*-Value		NS	NS	NS	NS

## Data Availability

The original contributions presented in the study are included in the article, further inquiries can be directed to the corresponding authors.
